# Interwoven urban crises: how health, housing, and climate change intersect

**DOI:** 10.3389/phrs.2026.1609638

**Published:** 2026-07-15

**Authors:** Ana Isabel Ribeiro, George Richard Lueddeke, Nino Kuenzli

**Affiliations:** 1 CEGOT—Centre of Studies in Geography and Spatial Planning, Faculty of Arts and Humanities of Porto, University of Porto, Porto, Portugal; 2 EPIUnit ITR, Institute of Public Health of the University Porto, University of Porto, Porto, Portugal; 3 One Health for One Planet Education and Transdisciplinary Research (1 HOPE-TDR), Southampton, United Kingdom; 4 SDSN Canada, Calgary, AB, Canada; 5 Earth Charter International (ECI), San José, Costa Rica; 6 Universities of Latin America and the Caribbean (UDUALC), Mexico City, Mexico; 7 Association of African Universities (AAU), Accra, Ghana; 8 Young European Research Universities Network (YERUN), Brussels, Belgium; 9 Department of Epidemiology and Public Health, Swiss Tropical and Public Health Institute, Basel, Switzerland; 10 University of Basel, Basel, Switzerland; 11 Swiss School of Public Health (SSPH+), Zürich, Switzerland

**Keywords:** climate adaptation, climate and health, gentrification, housing, neighborhood environments

Climate extremes are intensifying, and housing is becoming increasingly unaffordable. Today, 1.6 billion people still live without adequate shelter, and climate change is expected to cause 250,000 deaths each year by 2050. Cities are at the forefront of an interwoven crisis, facing severe housing shortages, widening health disparities, and escalating climate threats. Addressing these interconnected challenges requires a cohesive urban policy agenda that places climate-resilient, healthy, and affordable housing at its core.

Climate change has become one of the most pressing public health challenges of the 21st century [1]. Between 2000 and 2020, 3.39 million people died due to climate-related disasters, with heatwaves, tropical cyclones and floods being the top three deadliest [2]. The joint call for papers of IJPH and PHR “*Mitigating and adapting to climate change: evidence for public health”* pointed to a wide range of global public health issues amplified by and interlinked with climate change. While Schulte et al. addressed the direct impact of heat waves in a wealthy country, Gobena and Mengistu reviewed the indirect links between climate variability and food-borne diseases. Expanding the scope further, Amekpor et al. explored integrated initiatives to mitigate climate change with a focus on maternal and child health. In parallel, Akthar and Reid, along with Traoré and Tetka, investigated the broader challenges that climate change poses to health systems.

However, none of these studies addressed the interconnected crises of climate and housing. In fact, the figures above mask socioeconomic disparities deeply linked to the population’s housing conditions. For instance, during the 2003 heatwave in France, lack of thermal insulation and sleeping on top floors were associated with heightened mortality Traoré and Tetka. Similarly, in Barcelona, heat-related mortality was shown to be higher in census tracts with older buildings [3], underscoring how housing quality can amplify climate vulnerability.

Housing conditions shape the link between climate hazards and health, as people experience climate impacts primarily indoors [4]. People spend about 90% of their time inside buildings, mostly at home, and those most vulnerable to climate-related health risks, such as children, older adults, and individuals with disabilities, may remain indoors 100% of the time [5]. Poor insulation worsens heat stress during heat waves, while structural weaknesses increase risk during floods and hurricanes. Climate change also accelerates housing deterioration [4]. In this way, climate change acts as a “risk multiplier”, compounding health inequalities through substandard housing conditions [5].

As climate risks intensify, another crisis unfolds: the housing affordability crisis, pushing more people into unsafe living conditions. The financialization of real estate, the rise of mass tourism, and the expansion of the sharing economy have all contributed to rising housing costs, accelerating gentrification and undermining health and wellbeing [6]. In Europe, according to Eurostat, house prices have surged by 37% since 2010, with the steepest increase occurring between 2015 and 2021. Similar trends are emerging across the globe, even in the Global South, positioning gentrification as a growing planetary concern [7]. The housing crisis and climate risks are now deeply intertwined: soaring prices are pushing low- and middle-income residents into less ideal neighbourhoods and homes [8], many lacking adequate cooling, increasing their exposure to extreme heat and respiratory illnesses. As housing affordability decreases, many are left with no choice but to endure substandard living conditions that exacerbate health inequalities and climate vulnerability.

Given these realities, housing must be a cornerstone in climate change mitigation and adaptation. Sustainable and resilient housing, coupled with climate-conscious urban planning, is essential for withstanding extreme weather events, ensuring safe and healthy living conditions, and reducing environmental impact. This is especially urgent given that housing contributes significantly to global greenhouse gas emissions [9]. Adaptations, such as elevated foundations in flood-prone areas, enhanced insulation for temperature regulation, passive cooling, urban greening or climate shelters, as mentioned by Sanz-Mas et al., can reduce vulnerability while promoting sustainability [10]. Beyond reducing emissions and climate vulnerability, these measures can also generate important co-benefits by improving health and wellbeing, lowering household energy costs, and reducing social inequalities.

Yet, adding another layer of complexity, sustainable urban development can also exacerbate existing inequalities. In many urban contexts, green infrastructure and energy-efficient housing projects have fueled climate gentrification, displacing low-income residents under the guise of adaptation. This phenomenon occurs when environmental improvements increase property values, displacing long-term residents who can no longer afford to live in newly “climate-proofed” neighbourhoods [11]. In Miami, higher-elevation areas, historically home to low-income communities, are now targeted by wealthier populations moving away from flood-prone zones [12]. Similar trends appear in European cities, where greening projects, put into place to minimise exposure to climate extremes and other environmental harms, increase costs and displace residents [11]. This cycle leaves vulnerable populations in high-risk, low-resilience housing. As climate change drives demand for resilient homes, property values will increasingly reflect exposure and resilience, widening disparities as lower-income groups are pushed into riskier conditions. Yet, this challenge also presents an opportunity for innovation in urban governance, requiring climate adaptation plans that combine resilience measures with affordability protections, such as rent controls, inclusive housing policies and increasing urban residents' involvement in decision-making.

Finally, it is also important to underscore the unequal impacts of climate change, which disproportionately affect populations in the Global South, thereby highlighting the need for more globally inclusive perspectives in public health research. Worldwide, 1.6 billion people -mainly in the Global South–live in inadequate housing without basic services like water and sanitation. Around 60% of them, including 350–500 million children, live in slums [13]. Climate change affects cities differently: poorer nations face rising health risks, while economic inequality may worsen in wealthier countries. Addressing the climate crisis requires both emission reduction and adaptive strategies tailored to each urban context. Recognising the interconnectedness of all life in a shared environment fosters holistic approaches, including education (formal and non-formal), empowering communities, while transdisciplinary research [14] helps develop innovative, context-specific solutions aligned with sustainable development goals. Combining these efforts can promote more resilient, equitable, and sustainable cities worldwide.

In conclusion, climate change, housing, and the health and wellbeing of people and the planet are profoundly interconnected, with poor housing conditions exacerbating climate risks. Urban policymakers in both high-income and low- and middle-income countries must move beyond siloed approaches and recognise that housing, health, and climate are deeply interrelated crises. The future of cities – and indeed of all life-supporting habitats – depends on breaking these silos, shifting our worldview from human-centrism to ecocentrism [15], thereby optimising global sustainability through integrated policies and strategies that can make a difference to the planet before it’s too late.
